# The Scarcity of Resident Medical Officers

**Published:** 1913-09-06

**Authors:** 


### THE SCARCITY OF RESIDENT MEDICAL OFFICERS.

### The Facts, the Cause, and the Remedy.

Newly qualified medical men and hospital managers are equally concerned in the general difficulty which attends the filling of resident appointments in the hospitals unattached to medical schools, and from which even those institutions which have medical schools behind them are not wholly and invariably free. The medical student and the newly qualified medical man are, it is true, only too content to swim with the tide, in whatever direction fashion may point, as the readiest road to success in their profession, but that is no reason why they should not be placed in possession of the facts in order that their decision and policy at the opening of their professional careers may be based on something rather more solid than a thoughtless conventional usage. Indeed, the *raison d'être* of The Hospital's Special Educational Number is to place the facts on record that the student's choice may be an intelligent one, which, it should need no emphasis, is a matter of very urgent importance to institutional managers, who, in certain respects, are at the mercy of the young men who are about to form the class from which their resident appointments will be filled from this month onwards.

### The Present Position.

Both parties, the young resident class and the hospital managers, are only partially acquainted with the facts which information in our possession enables us with some confidence to summarise as follows. There is undoubtedly a scarcity of resident medical officers, which practically speaking all the county hospitals, and some of the larger city hospitals as well, are feeling. It is especially felt by those institutions which have less than one hundred beds. The first cause of this, which is sometimes much too carelessly put forward for accounting for the whole situation, is the apparently smaller entry into the medical profession which is the case to-day. The second cause is the complex one arising from certain special circumstances which have arisen only during the past few years. Taking these, rather in the order of their general recognition than in that of their importance, we find that the Insurance Act by the institution of panel practice has attracted many young men to its appointments by enabling them to start practice without the previously necessary possession of capital, and that young men, at the moment, can get "locums" at fees with which the voluntary hospitals, even in these days of greatly increased salaries for resident officers, cannot in practice compete. When panel practice has shaken down and its true advantages and disadvantages have been impressed by the hard school of practical experience upon a few "years" of medical men, the medical schools will hear less unconsidered reports than are current just now, and this difficulty thus created for the hospitals will probably cure itself.

There is, moreover, the attraction of "locums" to general practitioners, at sometimes five, or even more, guineas a week, which, especially in the holiday seasons of the year, proves overwhelming to newly qualified young men, naturally, if perhaps shortsightedly, only too anxious to get a quick and high return on the expenses of their medical education. Such young men often desire to skip the junior resident post altogether, saying that they would only be wasting their time as junior house surgeons, that their opportunities for surgical experience are too few, and even on occasion adding that the clinical teaching offered to them is inadequate. Another argument that is put forward as contra-indicating a hospital post is the young resident's dislike of visiting out-patients at their homes. At some hospitals, so persistent has been the scarcity of residents and so recurrent the objection to home visiting that it has even been abolished. Of course, home visiting often involves not very interesting visits to old people and chronic cases, and though these will form an important fraction of any general practice and therefore have much to teach the newly qualified in the art, if not in the science, of medicine, it is too difficult to persuade a man fresh from the schools of these facts. The home-visiting difficulty has even been acute enough in some places to produce a demand for a medical superintendent with one or more assistants on the ground that they would prove a preferable substitute for the honorary medical staff, who, it is said, as a class, are not always good at controlling young residents. We merely mention this as an instance of how galling the scarcity of residents and the home-visiting question have been known to prove.

### The Hospitals' Point of View.

Again the special circumstances conducing to a scarcity of residents include not merely the creation of panel appointments, and the attraction of locums to general practitioners, but the various other new openings to medical men which have arisen during the past few years. As the advantages and disadvantages of most of these are considered elsewhere in this Special Number, we will mention here merely the names of a few: the new tuberculosis appointments, the two Services, including the recent popularity of the Royal Army Medical Corps, the School Medical Service (dealt with fully in our issue of September 7, 1912), the foreign, colonial, and ships' doctor posts, the appointments under the Public Health and Poor-Law Acts, and so on. All these enter into competition with the resident posts of the voluntary hospitals.

The hospital side of these facts is that the smaller institutions feel that the numbers and quality of the men offering for their resident posts have decreased, and often where there used to be half-a-dozen applicants for an appointment there is only one to-day. Sometimes, indeed, where several applications for a post are received perhaps only one candidate will turn up on the day of election. So far, it would seem that special circumstances are responsible for the difficulty in which the hospitals are placed, for which, however, we shall see in a moment that the hospitals themselves are in part responsible.

In the face of this situation what have the hospitals actually done? The reply is a unanimous cry of protest: "We have raised the salaries to three figures and more." It may be said at once that this policy has postponed rather than solved the difficulty, which, however, in certain respects above explained is likely to be only a temporary one. But from the heart-searchng difficulties of obtaining suitable or any candidates one or two helpful hints can be gleaned.

### The Art of Advertising.

And, first, as to advertising. Replies to advertisements through the customary *media* have not been always satisfactory. Thus one or two facts become clear. The system of advertising these appointments which has obtained for so long must clearly be reconsidered. From the point of view of the medium it is clearly established that an advertisement buried among sixty or seventy pages of other advertisements, set in closely crowded columns, is not likely to be successful. Too many advertisements may be as uninforming as too few. The student simply will not be bothered with a haystack, where modern channels have provided an inviting mangerful of six rather than sixty pages, with two rather than three or four columns. As it was naturally the best administered hospital offices which were the first to realise this, the mangerful has proved of the best quality, for as we shall see fully in a minute the success of such advertisements also very largely depends upon the repute of a particular hospital at the various medical schools. Another point is this: It is important to arrange the term of the resident appointments, so that the vacancies fall in October and in April, when they will not interfere with the holidays, at which times the younger medical men have abundant opportunities of securing "locums" at fees actually known to have reached five or even as much as seven guineas a week.

### The Managers' Responsibility.

Coming now to the factor which the hospitals themselves have been in creating the scarcity, it may unhesitatingly be said that the best administered hospitals have the least difficulty. Much depends upon the quality of the medical and surgical work done in the institution. Able young medical men will not apply for resident posts at hospitals which are not well equipped technically and clinically, and generally administered on up-to-date modern methods. When the honorary staff of a hospital consists of general practitioners, and where the opportunities for surgical and scientific work are out of date, young qualified men, good enough and keen enough to desire to master their profession before seeking a cash return, avoid the place, and rightly so, for recent events have given forcible evidence that such hospitals exist, and are virtually asleep and inefficient. For, to state the truth, certain county hospitals neither find nor deserve to find it easy to secure resident officers, even if they can keep the resident posts filled continuously at all.

### A Patients' Strike.

The working men, whether industrial or agricultural, and the middle-class which is dependent on the voluntary hospitals in severe illness, are beginning to understand what a British hospital ought to be at the present time, and when this growing consciousness becomes general throughout the country, the above noted several sleepy hospitals, which are notably behind the times, will have to be revolutionised and swept of incompetence, or they will mercifully disappear. The residents in the locality will see to that by refusing to seek admission until the hospital's administration be reformed. For, it must be said as the serious moral to the whole question, that where it is difficult to obtain resident medical officers to-day it may be difficult to obtain patients to-morrow. So true is it, as we have always contended, that citizens in every locality deserve the hospital, good or bad, which they possess.

Hospitals, of course, are dependent upon local popular support, and where the medical and surgical arrangements are contemptible or out of date, where the nursing of a so-called training school (at which young women are kept for three years and not trained though often certificated), where there is an absence of pathological and other scientific means and equipment for the treatment and study of disease on modern lines, the fault lies with the hospital if residents prove unobtainable, and with the people if they experience the avoidable suffering which such an institution may bring to growing numbers of sick and helpless folk.

The real centre of control and supply of competent candidates for resident medical officers' appointments is undoubtedly the medical school. A qualified medical practitioner before applying for any resident post, will, if he is a wise man, ascertain from the Dean of his school, or from the senior men who have held these posts, the actual position of affairs in any particular hospital, so far as the duties and prospects of learning the practical side of his profession are concerned. Wise administrators, therefore, keep in touch with the Deans of the nearest medical school, or of that from which the majority of their honorary staff have come. This plan has two advantages: The honorary staff realise the difficulty, and consequently help to secure candidates through their personal influence at the schools.

